# The Efficacy of GnRHa Alone or in Combination with rhGH for the Treatment of Chinese Children with Central Precocious Puberty

**DOI:** 10.1038/srep24259

**Published:** 2016-04-13

**Authors:** Mengjie Wang, Youjie Zhang, Dan Lan, Jennifer W. Hill

**Affiliations:** 1 Center for Diabetes and Endocrine Research, Department of Physiology and Pharmacology, University of Toledo College of Medicine and Life Sciences, Toledo, Ohio 43614; 2 Center for Hypertension and Personalized Medicine, Department of Physiology and Pharmacology, University of Toledo College of Medicine and Life Sciences, Toledo, Ohio 43614; 3 Department of Pediatrics, the First Affiliated Hospital of Guangxi Medical University, Nanning, Guangxi, 530021, China; 4 Department of Obstetrics-Gynecology, University of Toledo College of Medicine and Life Sciences, Toledo, Ohio, 43614.

## Abstract

The addition of recombinant human growth hormone (rhGH) to GnRH agonist (GnRHa) to treat central precocious puberty (CPP) is controversial. We systemically reviewed and evaluated the efficacy and safety of the rhGH and GnRHa adjunctive therapy in Chinese children with CPP and assessed the influence of age and therapy duration on the efficacy of the combined treatment. A total of 464 patients were included from 14 studies. Compared with baseline, administration of GnRHa plus rhGH led to a significant increase in height, predicted adult height (PAH) and height standard deviation for bone age (HtSDS-BA), corresponding to a weighted mean difference (WMD) (95%CI) of 9.06 cm (6.41, 11.70), 6.5 cm (4.47, 8.52), and 0.86 (0.58, 1.14) respectively. Subgroup analysis showed the combined therapy had increased efficacy in subjects with initial treatment age younger than 10 years old or with treatment lasting over 12 months. Compared with GnRHa alone treatment, the combined treatment led to a significant increase in height, PAH and HtSDS-BA, corresponding to a WMD (95% CI) of 3.56 cm (2.54, 4.57), 3.76 cm (3.19, 4.34) and 0.56 (0.43, 0.69). The combined treatment exhibited no safety concerns. Our findings may aid clinicians in making treatment decisions for children with CPP.

Central precocious puberty (CPP) is defined as the premature activation of the hypothalamic-pituitary-gonadal axis with breast development before the age of 8 years in girls and an increase in testicular size in boys younger than 9 years old. CPP, also named gonadotropin releasing hormone (GnRH) dependent precocious puberty, is progressive and is often accompanied by advancement of bone age and accelerated linear growth^1^,^2^. In 2010, the prevalence of CPP was 55.9 per 100,000 girls in Asia with annual incidence of CPP ranging from 3.3 to 50.4 per 100,000^3^.

Gonadotropin releasing hormone analogs (GnRHa), the first-line treatment for CPP, briefly activates the hypothalamic-pituitary-gondal axis, but then suppresses the production of gonadotropin and consequent production of sex steroids. GnRHa use arrests pubertal progression, with variable regression of secondary sexual characteristics and decreased rates of linear growth and skeletal maturation, and consequently should improve final adult height^4^,^5^,^6^,^7^. However, several studies reported that GnRHa might also decrease height velocity below the age-appropriate normal range^8^,^9^,^10^,^11^. In some patients, the growth velocity (GV) decrease was so marked that it impaired predicted adult height (PAH)^12^. Detailed analyses of the growth hormone (GH)-insulin-like growth factor 1 (IGF-1) axis revealed that GnRHa treatment might decrease levels of free, biologically active IGF-1^13^,^14^,^15^. GH stimulates hepatic IGF-1 production and release into the circulation. To compensate for the reduced spontaneous or stimulated secretion of GH and IGF-1 during GnRHa therapy, it would be logical to add recombinant human GH (rhGH) in combination with GnRHa^9^,^16^,^17^.

In 1991, Oostdijk and colleagues firstly demonstrated that after 18 months of combined treatment of GnRHa and rhGH, 3 girls with CPP and low velocity obtained an improvement of PAH^18^. Volta and coworkers reviewed the combined therapy in CPP up to 2005 and demonstrated that this could benefit children with CPP and low GV to obtain a higher final height with a complete expression of genetic potential^19^. However, the Lawson Wilkins Pediatric Endocrine Society and the European Society for Pediatric Endocrinology suggested in 2009 that the addition of rhGH should not be recommended as a routine therapy due to lack of large-scale randomized, controlled trials evaluating the efficacy of the combined treatment^7^. Since then, little research has been done on the adjunctive treatment of GnRHa plus rhGH in children with CPP in western countries. In contrast, there are mounting studies investigating the efficacy of GnRHa and rhGH adjunctive treatment for patients with CPP in China since 2009. Several studies indicated that the combined treatment could significantly increase HtSDS-BA or PAH compared with the GnRHa alone^20^,^21^,^22^,^23^,^24^,^25^. However, their results were not consistent.

It is unknown whether the effects of GnRHa and rhGH adjunctive treatment on height are influenced by patients’ demographics, including age, and treatment duration. We therefore performed a systemic review and meta-analysis to evaluate the efficacy and safety of the addition of GH to GnRHa in treatment of Chinese children with CPP and to assess the influence of age and treatment duration on the efficacy of the combined treatment for CPP.

## Materials and Methods

### Search strategy

We performed “Medline, Embase, the Cochrane Library, Wanfang Database, VIP Database, and China National Knowledge Infrastructure” searches for all potentially eligible studies. The following free words were used: (gonadotropin releasing hormone analogs or GnRHa) AND (growth hormone or GH) AND (central precocious puberty or CPP) without any limitation. The bibliographies of retrieved randomized controlled trials (RCTs), case-control studies, observational studies, meta-analyses, narrative review articles, and relative references were reviewed by the authors. All reports were screened based on their abstract and references from general reviews were identified.

### Inclusion criteria

Citations selected from this initial search were screened for eligibility using the follow criteria: firstly, the study was RCT, case-control studies, or observational studies with at least one therapy arm consisting of GnRHa and GH adjunctive therapy; secondly, the patients were Chinese children with central precocious puberty characterized by the early maturation of the hypothalamic-pituitary-gonadal axis with breast development before the age of 8 years in girls and an increase in testicular size in boys younger than 9 years old^1^; lastly, the trial presented any outcome measure of height, PAH, or height standard deviation (HtSDS-BA) that could be included.

### Data extraction

The following data were extracted directly from the included trials: first author, year of publication, study arms, number of the patients, age, therapy duration, intervention, and outcome measurements (height, PAH and HtSDS-BA), body mass index (BMI), and insulin like growth factor-1 (IGF-1). Adverse effects and number of patients that dropped out of each study were also extracted. Mean and standard deviation (SD) were extracted for each outcome measurement. When the SD was not stated, we generated it from the sample size and the standard error or the 95% CI for each trials described by the Cochrane handbook for systematic review of interventions (version 5.0.1)^26^.

### Statistics

The weighted mean difference (WMD) for each continuous outcome was estimated to obtain a pooled measurement across studies. The WMD was first calculated as mean changes between the follow-up of the combined treatment and baseline, and then calculated between the combined treatment and GnRHa treatment alone. The effects of age and therapy duration on the overall estimates were assessed by meta-regression or stratification. Subgroup analyses were conducted by age (younger or older than 10 years old) and by therapy duration (shorter or longer than 12 months) to measure each outcome. Forest plots were generated to illustrate the study-specific effect sizes along with a 95% CI. Heterogeneity across studies was measured by the Q-test. *P* < 0.1 or *I*^*2*^ > 50% was considered to be significant heterogeneity^27^. The combined WMD was gained using random-effect model when the effects were assumed to be heterogeneous, otherwise the fixed-effects model was adopted^28^. Funnel plots as well as Egger’s test (if > 10 studies were included) were conducted to assess possible existing publication bias^29^. In addition, sensitivity analysis was performed to assess whether the overall meta-analysis estimate was influenced by any single study^30^. All analyses were conducted using STATA (version 11). P values were two-sided and significant when less than 0.05.

## Results

### Characteristics of trials

**Figure 1:**
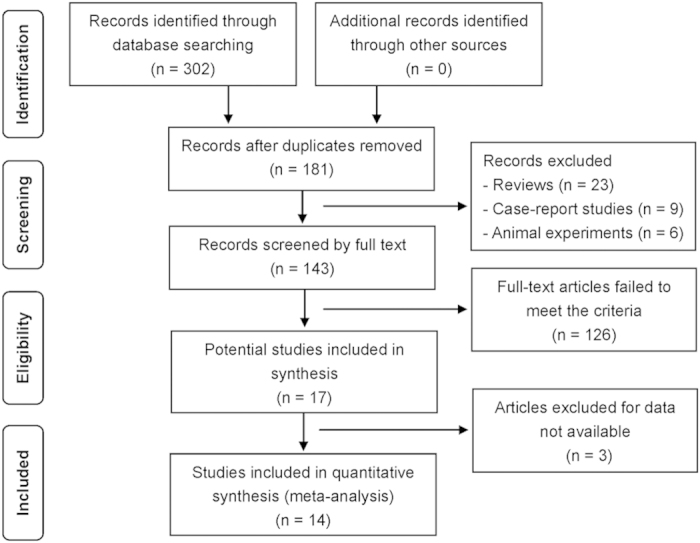
Flow chart showing the process of literature screening.

302 potential clinical trials were retrieved from published literatures; the trial selection process is presented in Fig. 1. Seven observational studies^31^,^32^,^33^,^34^,^35^,^36^,^37^ and seven case-control studies^20^,^21^,^22^,^23^,^24^,^25^,^38^ containing 464 patients that met the criteria were included in the analysis. Baseline demographic and clinical characteristics of the individual studies are shown in Table 1 and 2. All the patients were girls except for two boys included in the study by Wei and colleagues. The mean age in Table 1 was the initial treatment age. The age of onset of CPP, provided by some of the included studies, ranged from 6.2 to 7.8 years old^32^,^36^,^37^. The treatment duration of GnRHa alone before the addition of rhGH is mentioned in Table 1 if data was provided. Some of the included papers mentioned criteria for the addition of rhGH to GnRHa: firstly, GV was lower than 4 cm per year^25^,^33^,^34^,^36^,^38^; secondly, there was no obvious improvement in PAH^31^,^33^,^36^,^38^; thirdly, GnRH stimulation test found hypothalamus-pituitary-gonadal axis was inhibited by the GnRHa alone treatment^21^. Five different types of rhGH and three doses of rhGH were prescribed among all included studies: 4.5 IU/1.7 mg, 4 IU/1.33 mg, 16 IU/5.3 mg. However, the dosing guide was the same: 0.1 ~ 0.15 IU/kg*day (except Ma *et al*. prescribed 0.14 ~ 0.20 IU/kg*day), which means all the dosages ranged from 0.03 mg/kg*day to 0.05 mg/kg*day. Conversion between IU/kg and mg/kg was performed to facilitate comparisons between studies. Although the studies included were generally considered of good quality, none of the studies was a RCT.

### Outcome measurements

#### Effects of GnRHa plus rhGH adjunctive treatment for CPP

**Figure 2:**
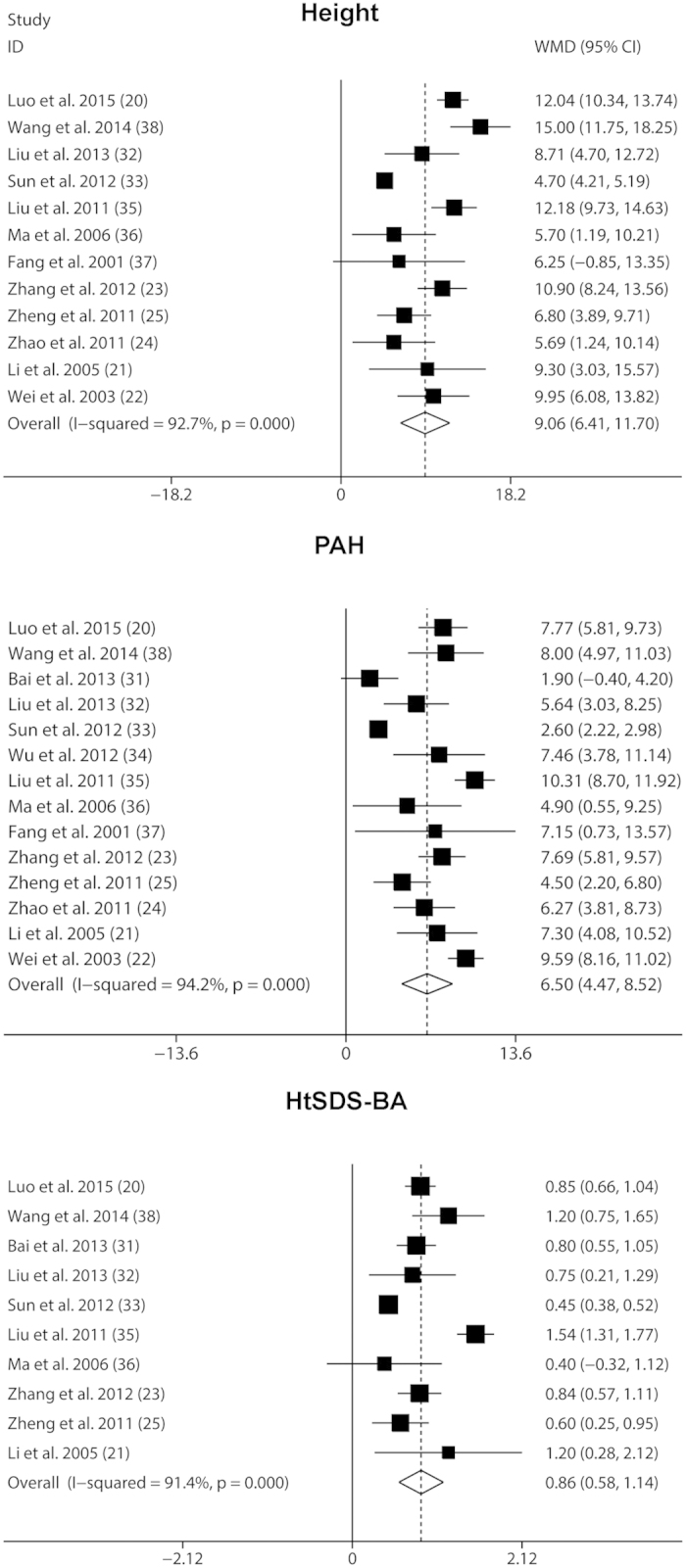
Random effects meta-analysis of GnRHa plus rhGH adjunctive treatment on Height, PAH and HtSDS-BA. The vertical line indicated no treatment effect. Squares and horizontal lines represent the point estimated with associated 95% CI for each comparison, respectively. Diamonds represent the random effects pooled WMD for each outcome. WMD, weighted mean difference; PAH, predicted adult height; HtSDS-BA, height standard deviation score for bone age.

A total of 306 patients that compared the follow-up of the GnRHa and rhGH adjunctive treatment and baseline were analyzed. After administration of GnRHa plus rhGH, a significant increase in height, PAH, and HtSDS-BA was found, corresponding to a WMD (95% CI) of 9.06 cm (6.41, 11.70), 6.50 cm (4.47, 8.52), and 0.86 (0.58, 1.14) respectively (all *P* values = 0.000) (Fig. 2). No long-term efficacy of the GnRHa and rhGH adjunctive treatment could be assessed due to the relatively short treatment duration among included studies. Nevertheless, we did an assessment after removing the shortest studies that had treatment duration of only 6 months. We found that compared with baseline, administration of GnRHa plus rhGH led to a more significant increase in height, PAH and HtSDS-BA, corresponding to a WMD (95% CI) of 10.38 cm (8.60–12.17), 6.94 cm (5.34–8.54), and 0.92 (0.68–1.17) respectively.

Meta-regression analyses were performed for separately investigating the influence of age and therapy duration on the change in each outcome measurement. Even though meta-regression did not show a statistical significance between the outcomes (height and HtSDS-BA) and subjects’ age, negative trends were seen between the outcomes and subjects’ age. A negative association was seen between the change in PAH and subjects’ age [β coefficient = −1.51, 95% CI (−2.67, −0.36), *P* = 0.015, n = 306]. In addition, meta-regression did not show a statistical significance between the outcomes (PAH and HtSDS-BA) and treatment duration, but positive trends were seen between the outcomes and duration. A positive association was seen between the change in height and treatment duration [β coefficient = 0.34, 95% CI (0.08, 0.61), *P* = 0.016, n = 261]. These findings indicated that the efficacy of the combined treatment might be improved as the patient’s age decreases or as the treatment duration increases.

In the subgroup analysis based on age, larger improvements were observed among patients with younger initial treatment age (<10 years old, as compared with >10 years old) with respect to height (10.19 vs. 4.71 cm) and PAH (7.60 vs. 2.60 cm) (Table 3). These findings indicated the combined therapy had increased efficacy in subjects younger than 10 years old. In the subgroups analysis based on therapy duration (12 months), larger improvements were observed between treatment lasting less than 12 months and over 12 months with respect to height (10.85 vs. 4.73 cm) and HtSDS-BA (0.98 vs. 0.45). These findings indicated the combined therapy had increased efficacy in subjects treated longer than 12 months (Table 3).

#### Effects of GnRHa plus rhGH vs. GnRHa alone for CPP

**Figure 3:**
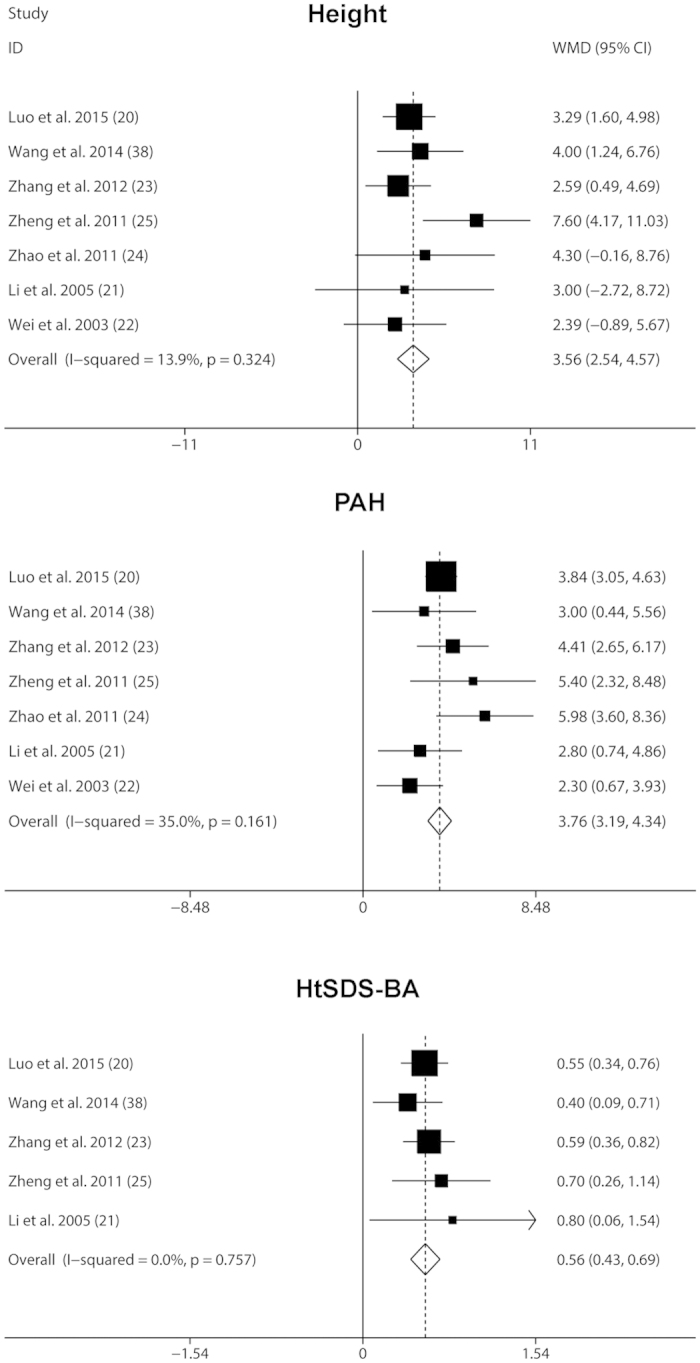
Fixed-effects meta-analysis of GnRHa plus rhGH vs. GnRHa alone treatment on Height, PAH and HtSDS-BA. The vertical line indicated no treatment effect. Squares and horizontal lines represent the point estimated with associated 95% CI for each comparison, respectively. Diamonds represent the random effects pooled WMD for each outcome. WMD, weighted mean difference; PAH, predicted adult height; HtSDS-BA, height standard deviation score for bone age.

To further evaluate the effect of rhGH on GnRHa treatment for CPP, we compared the combined treatment with GnRHa alone treatment. Seven case-control studies containing 142 patients in the combined group and 158 patients in the GnRHa alone group were analyzed. A fixed-effects model was used for analysis as we found there was no heterogeneity among the trials (all *P* values > 0.1 and *I*^*2*^ < 50%). Compared with GnRHa alone treatment, administration of GnRHa plus rhGH led to a significant increase in height, PAH and HtSDS-BA, corresponding to a WMD (95% CI) of 3.56 cm (2.54, 4.57), 3.76 cm (3.19, 4.34) and 0.56 (0.43, 0.69) respectively (Fig. 3).

#### Publication bias

Publication bias was assessed by Egger’s and Begg’s test. No publication bias was observed in the funnel plot of 14 studies evaluating the height, PAH and HtSDS-BA during the comparison of the combined treatment with GnRHa alone treatment. In the analysis of PAH changes for the combined treatment vs. GnRHa alone, the resultant funnel shape was asymmetrical, indicating publication bias (*P* = 0.008). Sensitivity analysis was also conducted to test if the results are sensitive to restrictions on the data included^30^. During the comparison of the combined treatment with baseline, the mean change in PAH was 6.50 cm (4.47, 8.52). The study of Sun and colleagues^33^ reported the largest variances in the change of PAH and the smallest increase in efficacy of the combined treatment vs. GnRHa alone treatment. Excluding this study from the analysis produced an estimate of change in PAH of 6.90 cm (5.47, 8.34).

#### Adverse effects include a statement about the adverse effects reviewed for both GnRH and rhGH

Among girls with CPP, GnRHa are generally well tolerated. Transitory adverse complaints, including headaches, hot flushes, depression, and irregular menses, occur occasionally^39^. However, these events do not prevent continuation of treatment. Local adverse effects such as erythema, induration, wheal and sterile abscess formation occur in less than 15% of patients^40^. Evidence shows that bone mass density (BMD) might be decreased after GnRHa treatment in patients with central precocious puberty and/or short stature. In 2007, van Gool and colleagues found that after 3-year rhGH plus GnRHa combined treatment, short adolescent boys showed a trend towards decreased BMD^41^. However, the sample size in this study was small (6 treated and 2 control) and the results were not significant. Lem and colleagues did not see adverse effects on the BMD and body composition in short children born small for gestational age after rhGH to GnRHa adjunctive treatment^42^. Other possible adverse effects of rhGH include an injection-site reaction, rare nerve, muscle or joint pain, and an increased risk of diabetes^43^. Direct and powerful evidence is lacking for long-term evaluation of the safety of the addition of rhGH to GnRHa in patients with CPP.

**Figure 4:**
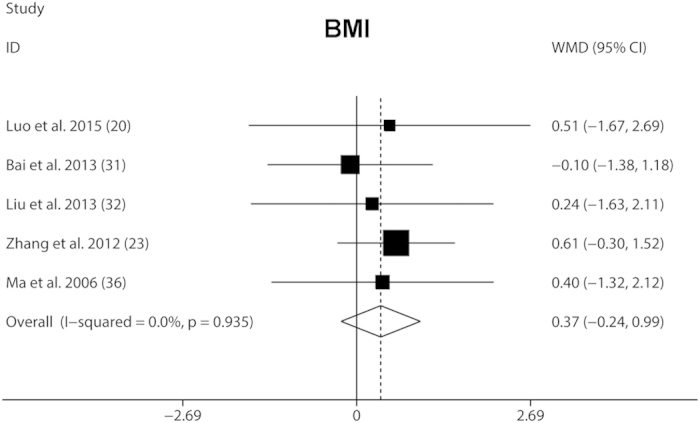
Fixed-effects meta-analysis of GnRHa plus rhGH adjunctive treatment on BMI. The vertical line indicated no treatment effect. Squares and horizontal lines represent the point estimated with associated 95% CI for each comparison, respectively. Diamonds represent the random effects pooled WMD for each outcome. WMD, weighted mean difference; BMI, body mass index.

To evaluate the safety of the GnRH plus rhGH treatment, we reviewed BMD, BMI, liver and kidney function tests, thyroid function tests, blood glucose tests, glycated hemoglobin Alc, insulin, blood tests, urine tests, x-ray of the hand and wrist, abdominal and pelvic cavity ultrasound, and magnetic resonance imaging or computed tomography for determining occurrence of tumors. All included studies found no obvious adverse effects during and after the GnRHa plus rhGH combined treatment, although the changes of BMD could not be evaluated due to lack of data among all included studies. No statistical significance for BMI was found during the combined treatment (Fig. 4). Compared with baseline, administration of GnRHa plus rhGH led to a significant increase in IGF-1, corresponding to a WMD (95% CI) of 39.31 ng/ml (30.77, 47.86). The increased level of IGF-1 after combined treatment confirmed previous detailed analyses of the GH-IGF-1 axis during the combined treatment^13^ and indicated that impaired height potential could be compensated for by the addition of rhGH to GnRHa. However, since only two studies showed IGF-1 levels after the treatment, we need more long-term evidence for the safety of the combined treatment.

## Discussion

Our meta-analysis shows a beneficial effect of GnRHa plus rhGH adjunctive therapy in Chinese children with CPP. The combined treatment was superior to either baseline or GnRHa treatment alone, corresponding to significant increases in height, PAH and/or HtSDS-BA. On subgroup analysis, the combined therapy had significantly increased efficacy in subjects with initial treatment age younger than 10 years old or with treatment lasting over 12 months. No apparent adverse effects occurred during the combined treatment in the published studies. This meta-analysis is the first report that the effects of GnRHa plus rhGH adjunctive therapy on outcome measurements (including height, PAH and/or HtSDS-BA) were influenced by patients’ age and treatment duration.

The efficacy of GnRHa plus rhGH adjunctive therapy has been previously described in the literature^16^,^44^,^45^ and is consistent with the hypothesis that rhGH compensates the reduced levels of GH and/or IGF-1, which results in final adult height increase. One recent study assessed height outcome between the GnRHa plus rhGH and placebo group in adolescents with relatively early puberty. Inconsistent with our results, they found that final height standard deviation score (SDS) [−2.0 (1.0) vs. −2.3 (0.6)] was not different between the combined treatment and the placebo group^41^. In our present meta-analysis, patients were exclusively diagnosed with CPP before the age of 8 years in girls. In contrast, van Gool and colleagues included adolescents with moderately early puberty (with Tanner stage 2–3, age less than 12 years for girls or less than 13 years for boys). Had we used a similar approach, it might have influenced the reported efficacy of the combined treatment.

For girls with CPP who show a marked decrease in GV, evidence indicates that the addition of rhGH to GnRHa is significantly more effective in improving height, and PAH than GnRHa alone^12^. One very recent study published in 2015 reported the combined treatment group showed a greater height increment during treatment in Korean girls compared to the GnRHa alone group^46^. In 2003, Pucarelli and colleagues^47^ found that the height gain in the combined treatment group was 6 cm higher than the GnRHa alone group. Final adult height in this study was defined as the height when growth was less than 1 cm/year with a bone age of over 15 years old^48^. Our meta-analysis showed that compared with GnRHa treatment alone, administration of GnRHa plus rhGH led to a significant increase in height and PAH of about 4 cm, lower than the findings from the study by Pucarelli and colleagues. This difference is likely due to the longer treatment duration (2–4 years) and long-term follow-up of their study than those included in our present meta-analysis. It may also be noted that two RCTs also reported final height in adopted girls with early or precocious puberty^49^,^50^. The differences in final height between the groups treated with GnRHa with or without rhGH were all around 3 cm. One meta-analysis published in 2014 by Li and coworkers pooled these two RCTs and focused on the comparison between the GnRHa and control group, in contrast to the objectives of our present meta-analysis^51^. Nevertheless, they also reported no significant difference in the final height SDS and initial height SDS when comparing the combined treatment group with GnRHa alone [pooled standard difference means = −0.28, 95% CI (−0.74, 0.19), *P* = 0.243]. However, according to our strict inclusion criteria, some girls included in these two studies (with early or precocious puberty with age between 6 to 12 years old) would not be diagnosed as CPP. Only Chinese girls younger than 8 years old with CPP were included in our meta-analysis. Finally, those studies were not stratified to assess associations between patients’ age and outcome measurements including height and/or PAH, as we have done.

On subgroups analysis, we found that the combined therapy had significantly increased efficacy in subjects with initial treatment age younger than 10 years old. Consistent with our findings, several studies also found that adult height outcome is influenced negatively by the age at which treatment starts^52^. The Lawson Wilkins Pediatric Endocrine Society and the European Society for Pediatric Endocrinology stated that the efficacy of GnRHa in increasing adult height is undisputed only in early-onset CPP for girls less than 6 years old^7^. In girls younger than 6 years old with onset of puberty, the greatest height gain reported was 9–10 cm^8^,^53^. The guidelines for the diagnosis and treatment of growth hormone deficiency suggested that these patients should be treated with rhGH as soon as possible^54^. Because of the very limited data discussing the efficacy in girls less than 6 years old, it was not possible to conduct subgroup analyses with age stratification at 6 years old. In most studies, the age of starting either GnRHa therapy of children with CPP, or rhGH therapy for children with growth hormone deficiency (GHD), was 3 to 4 years older than the “recommended” optimal age^7^, consistent with the mean age (9 years) of subjects included in the present meta-analysis. For the age groups included in this study, we found that girls less than 10 years old had higher height, PAH and HtSDS-BA at the end of the treatment.

We conducted further subgroup analyses based on the treatment duration (12 month) when enough studies were available. Larger improvements were seen in subjects with treatment lasting over 12 months as compared with treatment less than 12 months. Since the dose of GnRHa among all included studies ranged from 0.03 mg/kg*day to 0.05 mg/kg*day, it is not possible to perform subgroup analysis to determine whether the dose of GnRHa might also influence the efficacy. Although studies on GnRHa have accumulated over two decades, evidence of effects in boys is sparse in studies from both China and Western countries. Only two out of 464 subjects involved in our present meta-analysis were boys. We also lack data to test the psychological influence of the combined treatment of GnRHa plus rhGH on children with CPP.

Few adverse effects have been reported for rhGH and GnRHa combined treatment, though conclusions must be cautious because of the invasive and expensive nature of the treatment. The safety of co-administration of GnRHa plus rhGH on metabolic parameters and the hypothalamic-pituitary-gonadal axis was confirmed in one large cohort of patients with CPP with a long-term follow-up^47^. One recent study showed that combined treatment did not affect BMI, which was consistent with our findings [0.49 kg/m^2^ (−0.21, 1.20)]^41^. Additionally, hipbone mineral density was not significantly changed in the treated group when compared with the placebo group. Similarly, in our meta-analysis, few adverse effects were seen in included studies. The expensive nature of this treatment is also an important consideration in the clinical setting. Toumba and coworkers reported that girls with CPP reached their target height and had a total height gain of 5 cm. Each centimeter cost about €2700 ($3500) per patient^55^. However, we need more data to definitively evaluate the financial burden based on the therapeutic effect. Currently, the therapeutic strategy should be tailored to individual patients of CPP.

Three necessary and important diagnostic criteria of CPP were used by all included studies: firstly, early appearance of secondary sexual characteristics in girls before 8 years (boys before 9 years); secondly, GnRH stimulation test indicating LH >12 IU/L (LH > 25 IU/L in boys), and the ratio of LH/FSH between 0.6 to 1.0; lastly, enlarged gonads with pelvic ultrasound showing increased ovarian volume >1 ml and several ovarian follicle with diameter > 4 mm (enlarged testicular volume ≥ 4 ml in boys). The second and third criteria are critical to prevent unnecessary treatment of children with premature adrenarche or adiposity-related changes in the absence of awakening of the HPG axis. All included studies identified their patients as having idiopathic CPP except Fang’s study^37^. Limitations of the present study require these results to be interpreted with caution. While results on height, PAH and HtSDS-BA were reported, available studies lacked reports of final adult height. Since most of the eligible studies were of short treatment duration (≤ 2 years), the results may not persist with long-term follow-up. In addition, publication bias might contribute to underestimation of the effect size. Lastly, given that the included studies were case-control or observational studies, more powerful RCTs are needed to evaluate the efficacy of the combined treatment for CPP.

Animal studies raise the concern that increasing IGF-1 levels could further advance puberty. These studies suggest that IGF-1 regulates the precise temporal and spatial development of GnRH neurons and GnRH neurosecretion^56^. Male and female mice with the IGF-1R deleted in GnRH neurons demonstrated delayed pubertal development. With IGF-1 administration to prepubertal juveniles, puberty was advanced in control females, but not in females with the IGF-1R deleted in GnRH neurons, suggesting that activation of IGF-1 signaling within the GnRH neuron is a primary trigger for increasing GnRH release at puberty^57^. In contrast, IGF-1 administration was unable to advance puberty in control males. Additionally, a recent rat study demonstrated that IGF-1 could also regulate LH, and presumably GnRH, release by modulation of Kisspeptin and N-methyl-D-aspartate -mediated neurotransmission^58^. Further studies are needed to elucidate these mechanisms. While studies have not conclusively demonstrated that IGF-1 advances human puberty, in healthy girls, higher childhood IGF-1 levels predict earlier age at menarche^59^, and girls with CPP have increased IGF-1 levels compared with age, BA, and pubertal stage-matched healthy girls^60^. Nevertheless, in accord with findings of direct actions of IGF-1 on GnRH neurons and upstream neuronal targets, the current analysis shows that GnRHa treatment effectively prevents any advancement of puberty that elevated IGF-1 levels might otherwise induce.

## Conclusion

Better understanding of the efficacy and safety of the addition of rhGH to GnRHa in treatment of children with CPP will allow for improvements in final height, PAH, HtSDS-BA and the psychosocial care of such children. The present systemic review and meta-analysis provides evidence that the addition of rhGH to GnRHa in therapy for children with CPP is superior to either baseline or GnRHa treatment alone. The efficacy of the combined therapy is increased with use in subjects with initial treatment age younger than 10 years old, or with prolonged therapy duration. No obvious adverse effects occurred with respect to the published studies. These findings may aid clinicians in making treatment decisions in the treatment of children with CPP in this population and spur further investigation of the impact of the addition of rhGH to GnRHa. Ultimately, multi-center, long-term, and well-designed studies are needed to further understand the mechanism, the efficacy, and the safety of the addition of rhGH to GnRHa during CPP therapy.

## Additional Information

**How to cite this article**: Wang, M. *et al*. The Efficacy of GnRHa Alone or in Combination with rhGH for the Treatment of Chinese Children with Central Precocious Puberty. *Sci. Rep*. **6**, 24259; doi: 10.1038/srep24259 (2016).

## Figures and Tables

**Table 1:** Characteristics of seven observational studies included in the present meta-analysis.

First author Year	Study arms	No. of patients	Mean age (year)	Study duration (months)	Intervention	End points	Adverse effects
Bai *et al*.^31^	GnRHa + rhGH	26	10.5	17.3	GnRHa: 90–110 μg/(kg*28 days) for 3 month, then 60–80 μg/(kg*28 days); 6 month later add rhGH: 0.14 U/(kg*day)	PAH and HtSDS-BA	No
Liu *et al*.^32^	GnRHa + rhGH	11	8.8	12	GnRHa: 80–100 μg/kg for first time, then 60–80 μg/(kg*28 days); 3 month later add rhGH: 0.7–1 IU/kg for 6 days per week	Height, PAH and HtSDS-BA	No
Sun *et al*.^33^	GnRHa + rhGH	60	10.5	6	GnRHa: 100–120 μg/(kg*28 days) for first time, then 50–100 μg/(kg*28 days); rhGH: 0.3 mg/kg for 6 days per week	Height, PAH, and HtSDS-BA	No
Wu *et al*.^34^	GnRHa + rhGH	18	9.7	14	GnRHa: 80–100 μg/(kg*28 days), ≤ 3.75 mg; 6 month later add rhGH: 50–67 μg/(kg*day)	PAH	Unclear
Liu *et al*.^35^	GnRHa + rhGH	30	9.4	>12	GnRHa: 80–100 μg/(kg*14 days) for one month, then 80–100 μg/(kg*28 days), ≤ 3.75 mg; rhGH: 0.14–0.20 IU/(kg*day)	Height, PAH and HtSDS-BA	No
Ma *et al*.^36^	GnRHa + rhGH	15	10.5	7.8	GnRHa: 80–100 μg/(kg*14 days) for one month, then 60–80 μg/(kg*28 days); 6 month later add rhGH:1 U/kg for 6 to 7 days per week	Height, PAH and HtSDS-BA	No
Fang *et al*.^37^	GnRHa + rhGH	4	9.3	6	GnRHa: 3.75 mg/28 days; rhGH: 0.1 U/(kg*day)	Height and PAH	No

CPP: central precocious puberty; GnRHa: gonadotropin releasing hormone analog; rhGH: recombinant human growth hormone; PAH, predicted adult height; HtSDS-BA, height standard deviation score for bone age; BMI, body mass index; IGF-1, insulin like growth factor-1.

**Table 2:** Characteristics of seven case-control studies included in the present meta-analysis.

First author Year	Study arms	No. of patients	Mean age (years)	Study duration (months)	Intervention	End points	Adverse effects
Luo *et al*.^20^	GnRHa + rhGH GnRHa	30 30	8.5 8.5	18.7 19.6	GnRHa: 100 μg/(kg*28days) for first dose, then 60–90 μg/(kg*28 days); rhGH: 0.3 mg/kg for7 days per week	Height, PAH and HtSDS-BA	No
Wang *et al*.^38^	GnRHa + rhGH GnRHa	31 49	9.0 9.0	25.3 26.0	GnRHa: 105–106 ± 10 μg/(kg*28–35 days) for first dose, then ≤ 3.75 mg; 12 months later add rhGH: 0.14 ± 0.02 IU/(kg*day)	Height, PAH and HtSDS-BA	No
Zhang *et al*.^23^	GnRHa + rhGH GnRHa	20 20	8.4 8.3	20.2 17.4	GnRHa: 100 μg/(kg*28–32day) for first dose, then 60–90 μg/(kg*28 days); rhGH 0.3 mg/kg for 7 days per week	Height, PAH and HtSDS-BA	No
Zheng *et al*.^25^	GnRHa + rhGH GnRHa	26 23	9.1 9.5	30.8 22.3	GnRHa: 80–100 μg/(kg*28–32day) for first two times, then 60–80 μg/(kg*28 days); 19.4 ± 11.8 months later add rhGH: 1 U/kg for 6 to 7 days per week	Height, PAH, and HtSDS-BA	No
Zhao *et al*.^24^	GnRHa + rhGH GnRHa	15 15	9.4 9.3	6 6	GnRHa: 65 μg/(kg*28 days); rhGH: 0.15 U/(kg*day)	Height and PAH	No
Li *et al*.^21^	GnRHa + rhGH GnRHa	10 11	9.7 9.3	18.6 16.9	GnRHa: 100 μg/(kg*28–32days) for three months, then maintaining dose is 60–80 μg/(kg*28–32 days); 3 month later add rhGH: 0.23–0.28 mg/kg for 7 days per week	Height, PAH and HtSDS-BA	No
Wei *et al*.^22^	GnRHa + rhGH GnRHa	10 10	6.4 6.5	24 24	GnRHa: 100 μg/(kg*30 days) for three months, then use lower maintaining dose; 3 month later add rhGH:0.7–1 ug/kg for 7 days per week	Height and PAH	Unclear

CPP: central wprecocious puberty; GnRHa: gonadotropin releasing hormone analog; rhGH: recombinant human growth hormone; PAH, predicted adult height; HtSDS-BA, height standard deviation score for bone age; BMI, body mass index; IGF-1, insulin like growth factor-1.

**Table 3:** Results of meta-regression and subgroup analysis by age and therapy duration.

End points	Age/ therapy duration	No. of subjects	WMD (95% CI)	β coefficient (95% CI)	P value
Height		261		−1.40 (−3.41, 0.61)	0.153
	< 10 years old	187	10.19 (8.43, 11.94)		0.000
	≥ 10 years old	75	4.71(4.23, 5.19)		0.000
PAH		306		−1.51 (−2.67, −0.36)	0.015
	< 10 years old	205	7.60 (6.41, 8.78)		0.000
	≥ 10 years old	101	2.60 (2.23, 2.97)		0.000
HtSDS-BA		259		−0.13(−0.48, 0.22)	0.412
	< 10 years old	158	0.99 (0.70, 1.28)		0.000
	≥ 10 years old	101	0.57 (0.29, 0.86)		0.000
					
Height		261		0.34 (0.08, 0.61)	0.016
	< 12 months	94	4.73 (4.25, 5.21)		0.000
	≥ 12 months	168	10.85 (9.14, 12.56)		0.000
PAH		306		0.19 (−0.05, 0.42)	0.109
	< 12 months	105	4.85 (2.61, 7.10)		0.000
	≥ 12 months	201	7.23 (5.44, 9.02)		0.000
HtSDS-BA		259		0.04 (−0.01, 0.08)	0.093
	< 12 months	86	0.45 (0.39, 0.52)		0.000
	≥ 12 months	173	0.98 (0.72, 1.25)		0.000

PAH, predicted adult height; HtSDS-BA, height standard deviation score for bone age.
